# IoTTPS: Ensemble RKSVM Model-Based Internet of Things Threat Protection System

**DOI:** 10.3390/s23146379

**Published:** 2023-07-13

**Authors:** Urooj Akram, Wareesa Sharif, Mobeen Shahroz, Muhammad Faheem Mushtaq, Daniel Gavilanes Aray, Ernesto Bautista Thompson, Isabel de la Torre Diez, Sirojiddin Djuraev, Imran Ashraf

**Affiliations:** 1 Department of Artificial Intelligence, The Islamia University of Bahawalpur, Bahawalpur 63100, Punjab, Pakistan; urooj.akram@iub.edu.pk (U.A.); wareesa.sharif@iub.edu.pk (W.S.); mobeen.shahroz@iub.edu.pk (M.S.); 2 Higher Polytechnic School, Universidad Europea del Atlántico, Isabel Torres 21, 39011 Santander, Spain; daniel.gavilanes@uneatlantico.es (D.G.A.); ernesto.bautista@unini.edu.mx (E.B.T.); 3 Department of Projects, Universidade Internacional do Cuanza, Cuito EN250, Bié, Angola; 4 Research Group on Foods, Nutritional Biochemistry and Health, Fundación Universitaria Internacional de Colombia, Bogotá 11131, Colombia; 5 Universidad Internacional Iberoamericana, Campeche 24560, Mexico; 6 Universidad Internacional Iberoamericana Arecibo, Puerto Rico, PR 00613, USA; 7 Department of Signal Theory, Communications and Telematics Engineering, Unviersity of Valladolid, Paseo de Belén, 15, 47011 Valladolid, Spain; isator@tel.uva.es; 8 Department of Software Engineering, New Uzbekistan University, Tashkent 100007, Uzbekistan; s.djuraev@newuzbekistanuniversity.uz; 9 Department of Information and Communication Engineering, Yeungnam University, Gyeongsan 38541, Republic of Korea

**Keywords:** threat protection system, privacy, confidentiality, Internet of Things, machine learning

## Abstract

An Internet of Things (IoT) network is prone to many ways of threatening individuals. IoT sensors are lightweight, lack complicated security protocols, and face threats to privacy and confidentiality. Hackers can attack the IoT network and access personal information and confidential data for blackmailing, and negatively manipulate data. This study aims to propose an IoT threat protection system (IoTTPS) to protect the IoT network from threats using an ensemble model RKSVM, comprising a random forest (RF), K nearest neighbor (KNN), and support vector machine (SVM) model. The software-defined networks (SDN)-based IoT network datasets such as KDD cup 99, NSL-KDD, and CICIDS are used for threat detection based on machine learning. The experimental phase is conducted by using a decision tree (DT), logistic regression (LR), Naive Bayes (NB), RF, SVM, gradient boosting machine (GBM), KNN, and the proposed ensemble RKSVM model. Furthermore, performance is optimized by adding a grid search hyperparameter optimization technique with K-Fold cross-validation. As well as the NSL-KDD dataset, two other datasets, KDD and CIC-IDS 2017, are used to validate the performance. Classification accuracies of 99.7%, 99.3%, 99.7%, and 97.8% are obtained for DoS, Probe, U2R, and R2L attacks using the proposed ensemble RKSVM model using grid search and cross-fold validation. Experimental results demonstrate the superior performance of the proposed model for IoT threat detection.

## 1. Introduction

The Internet of Things (IoT) refers to a system of devices or objects connected to each other for data collection and sharing via the connected network [1]. The objects are embedded with sensors, software, and other useful technologies for connecting one device to another over the internet for the simple purpose of data sharing. The thing in the IoT can be any human with a heart organ monitor transplant [2], a biochip [3] transponder for animal monitoring on any farm, a sensor that provides alerts in automobiles [4] when the pressure of the tires is low, and other man-made or natural objects that are assigned internet protocol (IP) addresses in a network. Several performance evaluation metrics for IoT network performance have also been introduced to enhance the quality of service [5]. In the IoT, business and personal possibilities are boundless. In business, organizations and different industries use IoT systems to make their business handling and working more efficient, more reliable, with improved decision making [6]. The business values are also increased using these techniques.

The IoT has a significant role to play in the modern era. Using IoT sensors ensures different devices and objects around us are recognizable and locatable. The data consumed and transferred on the IoT may have significant information about people that is used in their daily lives [7]. The primary objective of the security of IoT devices is to prevent them from facing any risk and to keep the trust of users that their data are secure. Out of all the hurdles faced by the IoT, the significant one is the security challenge; particularly in the areas of personal privacy, the confidentiality of data in the domain of heterogeneous management, and network capacity limitations. The trustworthiness of security, its cost-effectiveness, performance efficiency, and privacy of the Internet of Things are keys for making sure that confidentiality, credibility, access control, and authentication are being maintained [7]. The IoT connects gadgets with people to communicate and lets them enjoy services. The majority of the devices that enjoy internet facilities nowadays lack basic security requirements, and hence, are prone to different security risks. IoT devices normally have memory limitations; hence, they use bandwidth communications that are very low in capacity. Present security mechanisms lack designs that support these limitations [8].

IoT devices are prone to several security challenges because they normally possess low computing power and complicated cryptography algorithms are not supported [9]. Currently, many experts confirm that the security of IoT devices is a major issue that we have to cope with if we want to adopt IoT gadgets securely. Different types of active and passive attacks are faced by the IoT network. In passive attacks, only information stealing is the target, while in active attacks [10], the attacker can physically damage the devices. In smart homes, there are many security threats such as continuous monitoring and the leakage of personal information, denial of service (DoS), distributed DoS (DDoS) attacks, falsification, etc. As IoT devices lack security mechanisms, they become easy targets. The further irony is that the victim has zero knowledge of being infected.

When it comes to IoT devices, attacks are broadly categorized into four groups; physical, network, software, and encryption. In a physical attack, IoT hardware devices are affected [11,12]. The network attack is launched in the IoT network. A software attack harms the software of an IoT system. The encryption attack works when the attacker breaks the security mechanism used in the IoT system. Thus, it is important to propose a security mechanism for devices according to device capabilities and characteristics. IoT device sensors collect physical quantities and convert them into a signal. Sensors also convert measurements from real-world environments into data. In the field of medicine, IoT devices are used to keep an eagle eye on patients. These devices can be attached internally or externally depending upon the situation or personal requirement. As IoT devices use wireless interfaces for communication, they are prone to cyber-attacks [13]. This leads to breaches in patients’ security. When an attack on medical devices is successful, it disrupts some fundamental applications that are providing surveillance on a patient’s critical medical condition. This may lead to patients losing their precious life. There are many kinds of attacks that a patient medical device (PMD) can endure, but the most familiar ones are eavesdropping, integrity error, battery-draining attacks, cyber security attacks, etc. An IoT device’s security can also be breached through social engineering attacks. Two of these methods include phishing and vishing. Social engineers hit the IoT the most because it includes human interaction. One of the most effective ways of avoiding and reducing the risk of social engineering attacks is enhancing the awareness of end users [14,15].

In IoT devices, there are plenty of loopholes as the IoT’s heterogeneous structure contains plenty of open weaknesses that are not fully managed [16]. The sensor and actuator layer is weak from direct physical access and there is a possibility that attacks such as DoS can break the security. The vast advancement in the services of the IoT requires an authentic and factual security mechanism. In devices, the simple sensor becomes very complex due to the vast need for devices and the enhancement in user requirements. The security can be enhanced by using a common IoT standard network, protocol, and commercially available IoT programming framework [17]. The security of IoT devices is easily penetrable because of simple security protocols and the lack of protective hardware [18]. Customers try to find their satisfaction in putting price before security when purchasing devices. Thus, customers have hardly any information about IoT devices for private and secure use [19]. There are several security mechanisms [20] available for security but user’s trust can still not be achieved. The existing authentication mechanism needs third-party approval. This type of authentication is considered under the risk of a single point of failure. The crucial data of authenticated devices can easily be altered by attacks along with maintaining anonymity [21]. To overcome loopholes of this nature, blockchain technology is used that is independent of third-party interference.

IoT devices are more susceptible to attacks due to most of the user’s interactions occurring in daily life, and these attacks can even be deadly. When these devices are attacked by an attacker, the user is also affected because the devices have many limitations for applying security mechanisms [20]. This research aims at developing the user’s trust by designing a proposed approach with effective machine learning techniques to prevent the threats [22]. The proposed approach uses machine learning models to identify threats in the IoT blockchain-based network. The network security laboratory knowledge data discovery (NSL-KDD) from the Canadian Institute of Cybersecurity is used in this study.

The major goal of this research is to build a system based on machine learning to increase threat detection accuracy. The performance of the individual machine learning models is analyzed in this research, but single models show poor performance. Instead of moving toward deep learning or more complex algorithms, the efficiency of the machine learning algorithms is enhanced using an ensemble approach. The main contributions of this research are as follows

- The proposed IoT threat protection system (IoTTPS) is based on an ensemble RKSVM machine learning model. The proposed approach enhances the threat detection accuracy in the IoT network and the security authentication.
- The machine learning models such as decision tree (DT), Naive Bayes (NB), logistic regression (LR), support vector machine (SVM), random forest (RF), gradient boosting (GB), K nearest neighbor (KNN), and proposed ensemble RKSVM model are used for experiments.
- The grid search hyperparameter optimization and cross-fold validation are used to enhance the performance of the ensemble RKSVM model and prediction accuracy.
- The evaluation parameters used to evaluate the performance of employed models include accuracy, precision, recall, and F1 score.
- In addition, performance comparison with state-of-the-art models is carried out. In addition, two datasets are used to validate the performance of the models.

The rest of the paper is organized as follows. Section 2 illustrates the efforts and technologies of the previous authors regarding IoT security. Section 3 shows the methodology, material, and methods that are used to carry out the experiments with the machine learning algorithms. Section 4 analyzes the results of experiments and illustrates the effectiveness of the proposed approach. Section 5 provides the conclusion of the proposed research.

## 2. Related Work

The IoT is one of the highest growing networks of those that are dependent on physical devices. These devices are capable of sensing and collecting data from the surrounding environment. Then different actions are performed according to the analysis results. These devices can also communicate with each other or with mainframe computers. The IoT brought an evolution in the domain of collecting real-time data. The IoT collects data by connecting things and devices that can share the data with the central machine, which provides flexibility to the data collection process that was not possible before the IoT [23]. This flexibility of the IoT to collect and monitor real-scenario environments facilitates numerous organizations or industries to communicate and connect to the information infrastructure through different sensors, things, and objects. This also adds significant value to different regions of the industry. The IoT also provides different significant benefits to the industries such as improved revenues, cost savings, task automation as well as possibilities to continuously bring innovation to the industry [24]. Similarly, the IoT presents tremendous opportunities for people and social economies to explore.

One of the most significant benefits of the IoT network is that the device and sub-IoT networks mesh together perfectly as a well-defined structure that presents the basis of any IoT architecture to enhance its effectiveness. In different circumstances, most of the IoT architecture solutions are related to the basic and fundamental issues that are connected to the IoT sensors, devices, connectivity, IoT platform, and eventually, the applications of the IoT [25]. The enhancements in the technology also allow the technology to add some additional layers to the architecture of the IoT [26]. The progressive characteristics of advanced technology are introducing new updates on a daily basis in the domain of the IoT, which makes it hard for researchers to follow up on a single architecture as a blueprint for concrete implementation. For that purpose, various reference architectures have been synchronized in the IoT field.

Every IoT solution [27] consists of specific IoT devices or sensors according to the situation. In any condition, when the IoT system is implemented for any specific situation, then the centralized system is also necessary to send data or receive the command from. IoT architecture is defined based on the integration with blockchain and software-defined networking (SDN) [28]. It allows the IoT networks to communicate with each other without interacting with centralized control devices. For broad and effective communication, all the IoT networks’ controllers are interconnected to each other in a distributed blockchain. When the attack begins, the packet migration components and analyzer of flow control are created to handle the network’s infrastructure’s main functions. The authors claim that the solution can detect any type of attack in the network quickly. To evaluate the proposed solution’s performance, the authors applied different attacks such as DDoS, fake topology, and ARP poisoning [29] individually and combined them to check the detection accuracy. The detection time of attack was reduced and the system provided faster network attack mitigation.

The availability of the data is the primary requirement in the IoT environment [30]. It also involves a large number of decision and analytic operations on the collected data in real-time scenarios. Such operations are prone to several threats to IoT networks. The intrusion detection systems in cyber security show great potential against IoT threats such as distributed denial of service (DDoS) and man-in-the-middle (MITM) attacks [31] based on the least square support vector machine (LS-SVM) by using sampling methods. For threat mitigation, efficient intrusion detection systems are needed. For example, threat mitigation for DDoS attacks is proposed by using SDN and specific IoT traffic features.

Edge computing was utilized in [32] by putting migrations and detection jobs to switches (open flow). A distributed approach to threat detection provides a fast response and detection of real-time attacks. Three machine learning algorithms are selected to segregate legitimate flows from DDoS attacks. The results show a superior performance from the RF model. SDN, fog computing, and machine learning techniques are used for threat detection in [33]. Recurrent neural networks (RNN), alternate decision trees (ADT), and multi-layer perceptron (MLP) are used to improve DDoS attack detection. The authors state that a fog computing-based solution shows good attack detection capability. The proposed security framework based on blockchain is used for IoT networks.

A blockchain-based approach for DDoS threat detection is proposed in [34] that uses the inter- and intra-domain migrations of DDoS. A multiple SDN-based domains scheme is allowed for secure collaboration and information on attacks is transferred in a decentralized manner. It allows operative attack extenuation near the base of the attack. The Naive Bayes machine learning algorithm is utilized for the intrusion detection system to protect the infrastructure of the IoT network from DDoS attacks. The multi-agent system based on the set of autonomous agents that can operate synchronously to receive and distribute experiences between IoT devices is used in [34]. The purpose of the actuator agent is to take action according to the found attack and disconnect the potential attacker from the network. Subsequently, the communication agent is employed to share information about intrusion detection in the network with other agents. The advanced methods can detect the intrusions and threats to the network very fast and efficiently, and also share the load to each participating agent very well.

The behavior of IoT-specific networks was analyzed in [35] and threat detection was enhanced using the feature selection process. Feature selection is carried out concerning the variant behavior of the network to enhance the accuracy of threat detection. The objective is to enhance the security of and prevent DDoS attacks on home networks. Machine learning models are employed for data acquisition, feature extraction, and binary classification for this threat detection system [36].

Study [37] employed statistical analysis to identify threats arising from endangered IoT devices. Nine IoT commercial devices were deliberately infected with the two broadly recognized IoT-based botnets, BASHLITE and Mirai. By using the mirroring port, the raw network traffic can be captured in the switch where the flow of traffic is very typical. The 115 features are obtained from the data of network-level packets. A neural network autoencoder was applied for threat detection for IoT devices. Eventually, the threshold for the threat was described as the sum of the sample mean and the standard deviation was calculated from the samples. A 100% true positive rate is reported in the study.

A tree ensemble model based on a greedy randomized adaptive search procedure using annealing randomness and feature selection was used to produce efficient results in [38]. The effectiveness was validated based on a secure water treatment testbed. An RF-based botnet detection was used and merged with three machine learning models [36]. The accelerated genetic algorithm with the rough set theory for the selection of efficient features was developed for the classification of threat detection. The authors present a hybrid ensemble model Catboost in [39] to deal with the security and intrusion detection problems of IoT networks. The model’s parameters are optimized using Bayesian optimization. Experimental results using the proposed approach show superior performance compared with existing models. A comparative summary of the discussed works is presented in Table 1.

## 3. Material and Methods

**Figure 1:**
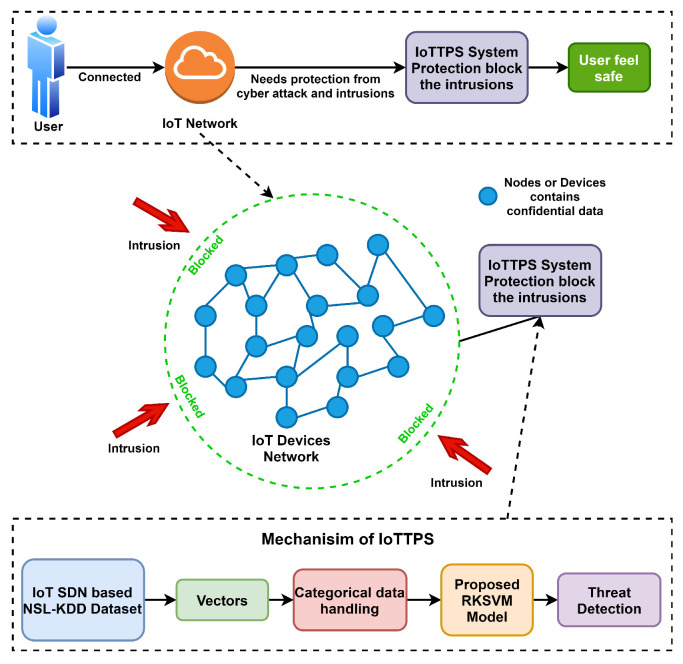
The workflow of threat protection IoTTPS system proposed based on RKSVM model.

This section presents the proposed approach, dataset, and models used for experiments. Figure 1 shows the workflow diagram of the proposed approach, IoTTPS for threat detection. This study adopts an ensemble model and the decision is based on the results reported in existing literature [39,48,49,50]. Often, ensemble models combining two or more models tend to show better performance than individual models.

### 3.1. NSL-KDD Dataset Presentation

**Figure 2:**
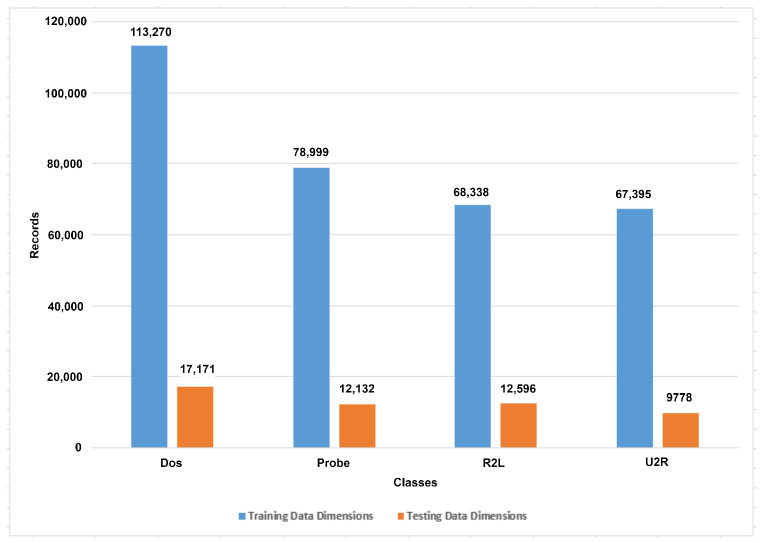
Dataset presentation according to the first top ten number of categories of threats and normal.

The IoTTPS system for IoT networks based on the ensemble learning algorithm is proposed in this research by using the NSL-KDD dataset. The NSL-KDD data were obtained from the well-known dataset repository Kaggle, which provides a wide range of benchmark datasets. The NSL-KDD dataset is split into training and testing subsets. The training data consist of 125,973 records and the testing data contain 22,544 records. The total dataset has 148,517 records for IoT network communication that contain intrusions-based data and normal data. A total of 23 categories of threats are presented in this dataset such as normal, neptune, satan, ipsweep, portsweep, smurf, nmap, back, teardrop, warezclient, pod, guess_ passwd, buffer_overflow, warezmaster, land, imap, rootkit, loadmodule, ftp_write, multihop, phf, perl, spy as shown in Figure 2. The neptune class contains the highest number of 41,214 records. The normal class presents the normal communication that contains 67,343 records. Table 2 presents the mapping to numerical values against classes.

These 23 categories are further converted into five major categories such as ’Normal’, ’Dos’, ’Probe’, ’U2R’, and ’R2L’, as shown in Figure 2. This reduces the number of classes and increases the efficiency of the ML model. The training data associated with the DoS class have 113,270 records with 123 attributes, the Probe class has 78,999, R2L class has 68,338, and USR class has 7395 records to train the ML models. Other details for training and test data are presented in Table 3.

**Figure 3:**
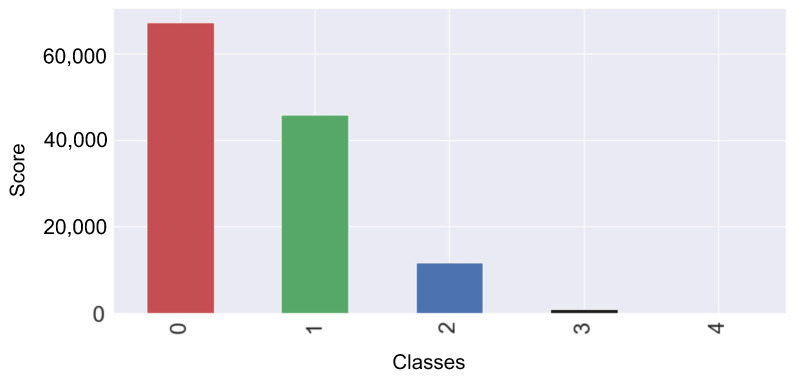
After the conversion of categorical data into numeric form.

The null values are removed from the dataset. The dataset contains the categorical values that cause issues for models’ training. The one hot encoder technique is used to convert the categorical data into numeric form to resolve this problem. The protocol_type, services, and flag are the categorical attributes. In the protocol_type, two types of protocols are used: transmission control protocol (TCP) and user datagram protocol (UDP), which are encoded into 1 and 2, respectively. Four types of services are used in the dataset such as ftp_data, other, private, and hypertext transfer protocol (HTTP), which are encoded into 20, 44, 49, and 24, respectively. Two types of flags are used such as SF and S0, which are encoded as 9 and 5, respectively. Figure 3 shows the number of samples for each class.

The KDD Cup 1999 dataset was created for the Third International Knowledge Discovery and Data Mining Tools Competition held in 1999. It contains 4 million labeled records divided into four classes: DoS (Denial of Service), Probe, R2L (Remote-to-Local), and U2R (User-to-Root) and includes 41 features, which are derived from IoT network connections and other relevant information. The NSLKDD dataset is the improved version of the KDD dataset by overcoming the limitations of the KDD dataset. The NSLKDD dataset consists of 1.8 million labeled records categorized into four classes: DoS, Probe, R2L, and U2R, providing a more balanced distribution of attack types than KDD. The CICIDS dataset created by the Canadian Institute for Cybersecurity is a more current intrusion detection dataset. The dataset contains 3.5 million instances including DoS, DDoS (Distributed Denial of Service), and probing attacks based on 79 features.

### 3.2. Methodology

**Figure 4:**
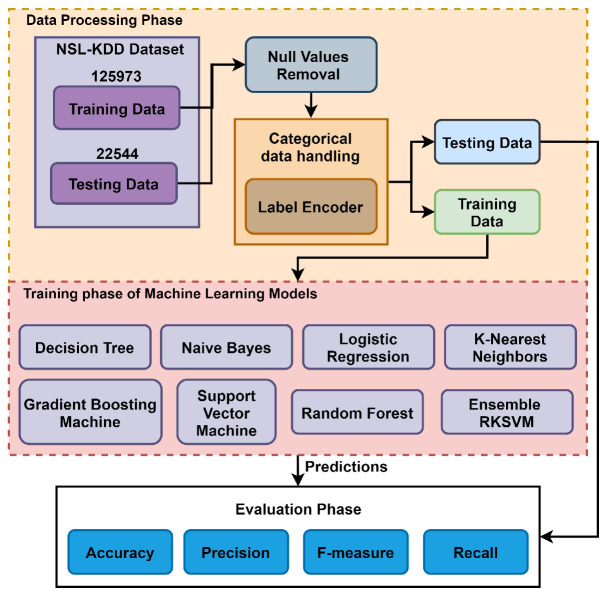
The mechanism of presented IoTTPS system based on machine learning.

The dataset is used as the vectors for the training of the model. For training, 125,973 records are used, which further contain different numbers of samples for each class. For example, for the major classes DoS has 113,270, Probe has 78,999, R2L has 68,338 and U2R has 67,395 records. The ML models are applied according to DoS, Probe, R2L, and U2R with the normal class. After the removal of null values and handling of categorical attributes, the dataset is ready for the ML models. The ML models include DT, NB, LR, SVM, RF, GBM, KNN, and ensemble RKSVM. Figure 4 presents the architecture of the adopted methodology of the proposed IoTTPS system. The performance of the ML model is evaluated using accuracy, precision, recall, and F1 measure.

#### 3.2.1. Decision Tree

DT is a non-parametric supervised learning method that recursively partitions the given dataset of rows by applying the depth-first greedy method [51] or the breadth-first approach [52] until all the data are related to an appropriate class. A DT structure is created from the root, internal, and leaf nodes. The tree construction is used in classifying the unknown data. At each inner node of the tree, a decision of best separation is made using impurity measures [53]. The DT classification procedure is implemented in two stages: tree building and tree pruning. The first stage is the tree building, which is performed in a top-down manner. During this phase, the tree is recursively partitioned till all the data objects relate to the same class label [51]. DT is computationally fast as the training dataset is traversed frequently.

(1) Informationgain(T,X)=Entropy(T)−Entropy(T,X)

(2) $$Gainratio=\frac{information\;Gain}{SplitInfo}$$

(3) $$Gainratio=\frac{Entropy\left(before\right)-\sum_{j-1}^{K}Entropy\left(j,after\right)}{\sum_{j-1}^{K}w^{j}log_{2}w_{j}}$$

where *T* presents the column and *X* presents the class variable. Entropy shows the calculated weight of the single attribute value and *Information Gain* shows the total calculated weight for which column is best to be a node in the tree.

DT classifies the occurrence by sorting them on the base of feature values. Each node in a DT represents a feature in an instance (classes such as 0 or 1) to be classified, and each branch represents a value (0 or 1) that the node can assume. The occurrences are classified starting from the root node and sorted based on their feature values, which are given in the form of classes [54].

#### 3.2.2. Support Vector Machine

SVM is a classification algorithm that utilizes the concept of a hyperplane that separates the classes. In other words, the algorithm outputs are based on an optimal hyperplane that categorizes new test data on the basis of training data. SVM can be used for both classification and regression. However, it is mostly used in classification problems, where it gives the best accuracy between the two classes. In this algorithm, each data item is plotted as a point in *n*-dimensional space, where *n* is the number of features in the dataset with the value of each feature being the value of a particular coordinate. Then, classification is performed by finding the hyperplane that differentiates the two classes very well.

SVM contains different tuning parameters but some are used that give the best accuracy at the countvectorizer and TF/IDF feature extractor. The tuning parameters of the SVM are used when the feature extractor is a countvectorizer *C*, which is adjusted from 1.0 to 1.1 to achieve better accuracy. Other parameters include gamma = auto, probability = False, and tol = 2, which is used for the stopping criterion. The class-weight = None, verbose = False, which enables verbose output, and the maximum number of iterations is 55. These parameters provide the best accuracy.

#### 3.2.3. Gradient Boosting Machine

GBM is a well-known tree-based algorithm that assembles multiple machine learning algorithms together and improves the accuracy based on boosting and bagging concepts. The GBM is commonly based on the ensemble of multiple decision tree models and applied for both classification and regression. These problems are solved by the GBM because the multiple decision tree architecture makes the complex gradient patterns easier for GBM. The GBM model gives different accuracies on different feature engineering techniques.

Several parameters are fine-tuned to achieve better accuracy. The n_estimators is set to 150, which presents the total number of stages of boosting performed by the GBM model, which enhances the performance and prediction accuracy of the model. The max_depth = 10 is the maximum number of levels of a tree that also controls the generation of nodes in the tree. The learning rate is set to 0.01 to reduce the contribution of each tree by the learning_rate parameter, and there is an adjustment between learning_rate and n_estimators. The tuning parameters that give the highest accuracy are n_estimators = 100, max_depth = 12, and learning_rate = 0.01.

#### 3.2.4. Random Forest

The RF classifier is used for threat detection [55]. RF algorithm gives different accuracies on different feature engineering techniques. The RF classifier uses multiple DTs as base classifiers [56]. The randomization in the RF model is built on two concepts: in the first approach the samples are extracted from the dataset randomly as these samples are collected in boosting concept of GBM [57]. The second one is to select the input features randomly for creating DTs as a base of the model. RF is optimized concerning different parameters such as n estimators = 13, here *n* indicates the number of trees in the forest and the range of the number of trees is from 10 to 100 and maxdepth = 150. The maxdepth indicates the maximum depth of the tree. The criterion parameters can have Gini or entropy to measure the quality of a split. When the criterion is Gini, then it is used for Gini impurity, and when it uses entropy, then it measures the information gain.

#### 3.2.5. Naive Bayes

NB classifier is one of the simplest and most effective machine learning classification algorithms, which helps in building a fast machine learning classifier that can make quick predictions from the given dataset. It is a probabilistic classifier, which means it predicts based on the probability of an object. Bayes algorithm is a probabilistic classifier that is built upon the Bayes theorem such as

(4) $$P\left(A|B\right)=\frac{\left(P(B|A)*P(A)\right)}{\left(P(B)\right)}$$

where *A* is the class, *B* is the feature vector, P(B|A), P(A), and P(B) are the probabilities measured from earlier known instances, such as training data.

The classification errors are minimized by selecting the class that maximizes the probability P(A|B) for every occurrence [58]. The NB classifier is considered to perform optimally when the features are independent of each other, and close to optimal when the features are slightly dependent [59]. NB performs well even if the dependency is clear between the features, making it fit a wide range of tasks.

#### 3.2.6. K-Nearest Neighbors Classifier

The KNN classifier is used for both regression and discrete data-based classification in machine learning. The data points are extracted from the training data and are classified based on the distance function also known as similarity index. KNN performs the classification operation based on the majority of the voting of its neighboring data points [60]. KNN model is also known as a slow learner, as it needs all instances for model training. It is necessary for the KNN model that all classes are populated with a balanced number of the dataset samples because the majority of data in one class gets priority. It is a simple approach; however, it can often perform better than complicated models.

#### 3.2.7. Proposed Ensemble RKSVM Model

**Figure 5:**
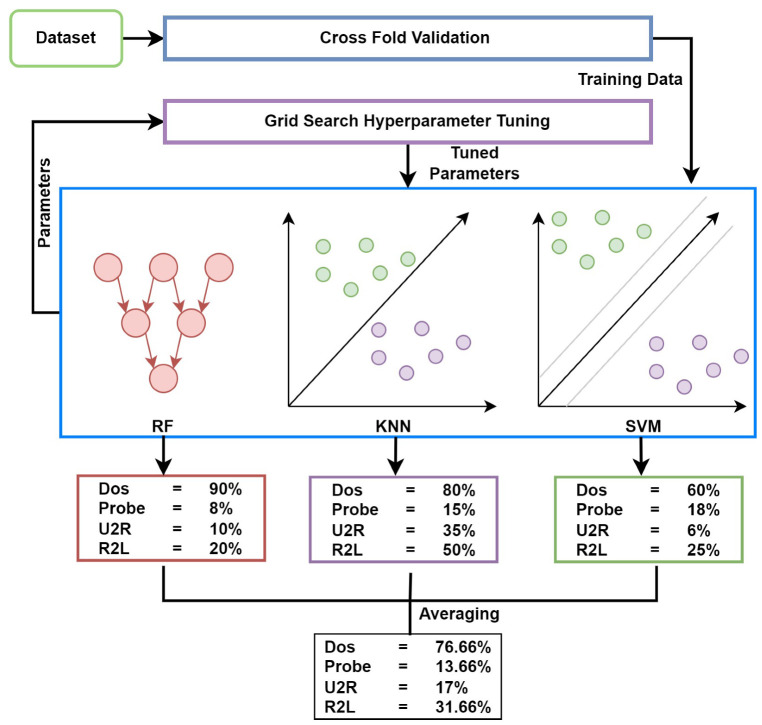
The architecture of the proposed ensemble RKSVM model.

The voting classifier is the simplest form of joining different classification algorithms in which selecting the combination rule is important for designing an ensemble classifier. Voting is the way of combining predictions from multiple machine learning algorithms [61]. In the proposed ensemble model, the voting scheme combines the predictions of three algorithms RF, SVM, and KNN. The voting-based ensemble was implemented using soft voting criteria. The experiments were performed with a soft voting technique and the averaging mechanism was considered for the prediction of every model used in the ensemble method. The experiments were conducted based on several combinations of machine learning models to analyze the performance of individual models. RF, KNN, and SVM were selected on the basis of their architectural design and after evaluation of the best results illustrated in the experiments. The RF depends on multiple decision trees that generate separate decisions and then selects the majority decision of trees. The KNN depends on K-nearest points as a neighbor and performs classification based on a similarity score. The SVM is based on a hyperplane that draws the hyperplane between different classes based on marginal space from the nearest data point to the hyperplane. The experiments showed these three models gained the highest results. These algorithms gave the best accuracy compared with others so these three algorithms were used for ensemble using voting, as shown in Figure 5. The cross-fold validation was used here to select the best dataset split ratio for training and testing data. The grid search hyperparameter tuning method was used for the tuning of the three selected models’, RF, KNN, and SVM, parameters to obtain optimized performance.

The grid search is the hyperparameter optimization technique used to tune the parameters of the machine learning model. The grid search takes input as manually defined parameter values for the model in an array. Then the model defines a pipeline for grid search to train one by one using those parameter values, which are defined by the user. The grid search selects one value for one parameter and then changes other parameter values with the same parameter. then one by one it creates a huge set of parametric values to train the model by using all sets of parametric values to obtain the best single set.

Cross-fold validation is applied to the dataset to obtain the best partitioning ratio for the train data and test data. The cross-fold validation was used here with the grid search optimization technique. Here cross-fold validation obtained the best training and testing data partitions from the original dataset. Grid search optimization was used to obtain the best model hyperparameters to train the model. Both these techniques with the ensemble of models created an effective and efficient model to detect cyber attacks.

The voting classifier with the stacking method was used and three classifiers were used for this voting purpose, SVM, RF, and KNN, which performed well and gave the highest accuracy. In these methods, the driving policy is to build several estimators separately and then take the average of their predictions. On average, the mixed estimator is normally better than any of the single base estimators as its variation is reduced [61].

The models used in the proposed ensemble were fine-tuned to find the best-fit parameters for obtaining optimal performance. The used parameters for three models are given in Table 4.

### 3.3. Performance Evaluation Metrics

The classification of threats detection by using the NSL-KDD dataset is based on the communication record of the IoT networks. Several machine learning algorithms are proposed in this research that help to detect the threats in IoT networks. The performance of these machine learning algorithms was evaluated using evaluation parameters such as accuracy, precision, recall, and F1-score.

The accuracy is dependent on the total number of predictions that are correctly made by the models from all predictions and is calculated using Equation (5).

(5) $$Accuracy=\frac{TN+TP}{TP+FN+TN+FP}$$

where TP, TN, FP, and FN represent true positive, true negative, false positive, and false negative instances predicted by a model, respectively.

The recall is the number of TP divided by the number of TP plus FN. The highest value of the score is 1, and the lowest value is 0.

(6) $$Recall=\frac{TP}{TP+FN}$$

Precision indicates the capability of a model to predict positive cases and is calculated using TP divided by TP plus FP. The highest value of the score is 1, and the lowest value is 0.

(7) $$Precision=\frac{TP}{FP+TP}$$

F1 score is regarded as a more suitable performance evaluation metric as it considers both precision and recall. It is more important than precision and recall, especially in situations where the dataset is imbalanced and the model may experience overfitting. It is calculated using

(8) $$F1score=2*\frac{Recall*Precision}{Recall+Precision}$$

Detection time presents the prediction rate of the model indicating how much time (seconds) a model takes to detect threats and normal communication from the testing data and is given in Table 3.

The throughput presents how many threats are detected by a machine learning model in a unit time (second).

(9) $$Throughput=\frac{Numberofthreatsdetected}{timeperiod}$$

The latency shows the time (seconds) taken by the machine learning models to predict a single attack.

## 4. Results and Discussion

The classification of threats related to SDN networks is proposed in this research. The NSL-KDD dataset was used for experiments that consist of 148,517 records related to different threats of 23 categories. As well as the proposed RKSVM approach for threat detection, several well-known machine learning models were employed in this research. For experiments, the models were implemented using the SciKit-learn library of Python. Experiments were performed using an Intel Core i7 machine running on Windows 20 operating system and 16GM RAM.

### 4.1. Experimental Results

The experiments were carried out using the ML models to analyze their performance concerning network threat detection. The results of the proposed approach and other machine learning models are given in Table 5. The highest accuracy for predicting different types of attack varies between 97% and 99%, and different machine learning models show different levels of performance regarding each threat category. The performances of the models are better in the DoS and U2R classes. DoS attack is detected with the highest accuracy of 99.7% from both the proposed approach and KNN and RF. Similarly, U2R is detected with an accuracy of 99.7% from RKSVM and KNN.

The performances of models concerning precision are shown in Table 6. The results demonstrate that, similar to the accuracy results, precision also varies with respect to each class and each model. On average, the results for the DoS and Probe classes are better where precision is concerned. Although the performance of RF and KNN is better, compared to other models used in this study, the highest precision is obtained by the proposed approach, except for RF, which achieved the best precision of 96.3% for the R2L class.

The recall results for all models are provided in Table 7, which indicates the superior performance of the proposed approach. All models seem to perform better, except for NB and DT, which show poor recall results for the DoS and Probe classes in particular. Overall, the proposed approach obtains better recall results for DoS, Probe, and R2L with a recall higher than 96%. However, recall for U2R is 86.5%, indicating a higher number of FN for this class.

The F1 score has been regarded as an important performance evaluation metric as it takes into account both precision and recall and can show the performance of the model regarding FN and FP. Table 8 shows class-wise results of all employed models. While the performance of the models is good for the DoS, Probe, and R2L classes, the F1 score for the U2R class is only 89.1%. This is the highest F1 score for this class achieved by the proposed approach, and the other models have a low F1 score with 87.8% as the best from KNN. Although the U2R class has a number of records similar to R2L, detection performance for this class is low. For the other classes, the proposed model performs better, with the highest F1 score, except for R2L, where RF obtains the best F1 score of 96.9%.

**Figure 6:**
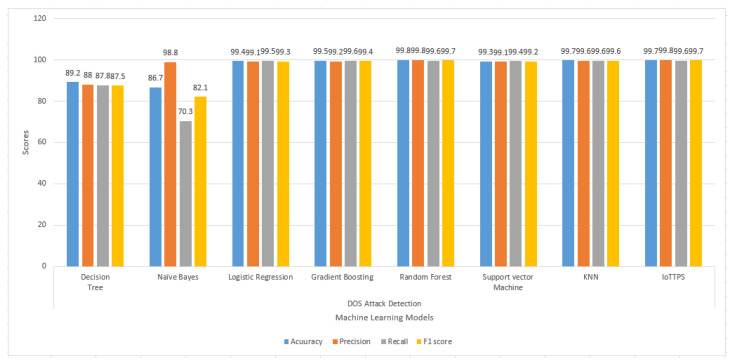
Experimental results of machine learning models to detect DoS attacks.

Class-wise results are also presented to analyze the performance of the machine learning models for each type of attack. Figure 6 illustrates the results of the machine learning models regarding the accuracy, precision, recall, and F1 score for DoS attacks. On average, all models perform well for DoS attack, except for DT and NB, which show lower scores for accuracy, recall, and F1 score. The best performance is obtained by the proposed approach for DoS attacks with better accuracy, precision, and other metrics.

**Figure 7:**
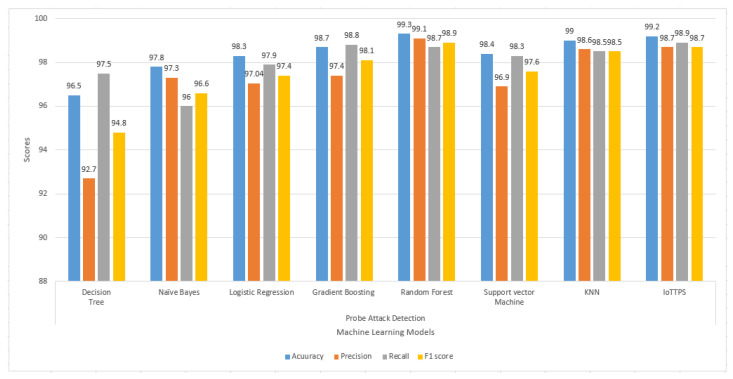
Experimental results of machine learning models to detect Probe attacks.

Experimental results for the Probe class for accuracy, precision, recall, and F1 scores are given in Figure 7. For this attack, the performance of the models varies significantly regarding all parameters. RF and the proposed approach show the best results for Probe attack detection. DT and NB again show poor performance, especially concerning precision and recall, respectively. The lowest precision and F1 scores are obtained from DT. Only RF, KNN, and the proposed approach provide higher than 90% results for accuracy, precision, recall, and F1 score.

**Figure 8:**
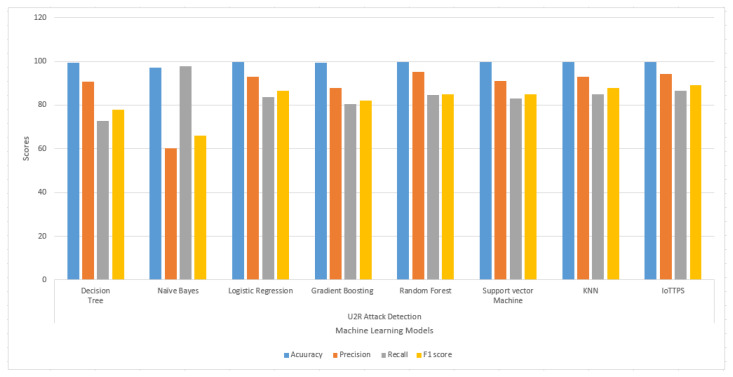
Experimental results of machine learning models to detect U2R attacks.

**Figure 9:**
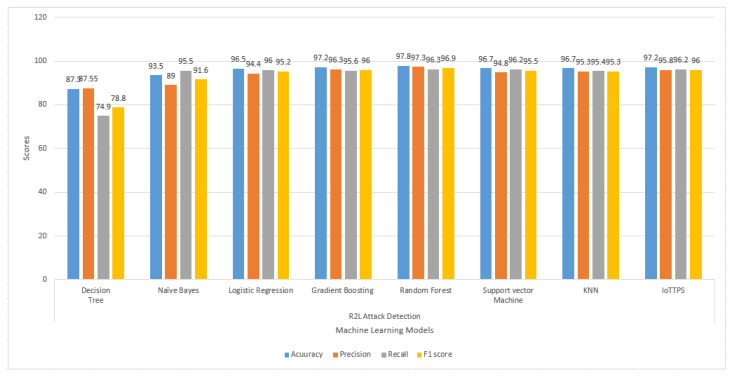
Experimental results of machine learning models to detect R2L attacks.

The results regarding the U2R and R2L attacks are presented in Figure 8 and Figure 9, respectively. The results indicate that the performances of the models are substantially degraded for U2R attack detection. The overall scores for the evaluation parameters are higher than 80% for all models except for DT and NB. NB shows the poorest precision and F1 score, while the recall and F1 scores of DT are also lower than other models. For R2L attacks, the performance of the models is better; even DT and NB perform well except for recall and F1 score from DT, which is comparatively lower. The proposed model performs well compared to the other employed models.

### 4.2. Discussions

DT is a tree-based model that consists of a number of nodes that contain data, a leaf that contains final classification results, and a weighted link that connects the nodes. DT does not perform well with the NSL-KDD dataset because it is sensitive to perturbations and changes in the data can significantly affect tree formation. Similarly, DT can easily overfit. The highest accuracy of 89.2% by DT is for the DoS class, which is lower than the other models. For the Probe class, it obtains 96.5% accuracy, 92.71% precision, 97.5% recall, and an F1 score of 94.84%. Its result for U2R is also better. However, for the R2L class, it obtains an accuracy of 87.3%, precision of 87.55%, recall of 74.94%, and F1 score of 78.80%, which are lower.

The GBM is based on the concepts of bagging and boosting where several iterations are performed by the GBM to extract the patterns from the dataset. The GBM randomly selects the samples from the record and the remaining samples are reserved for further iterations that probably increase the prediction accuracy. It shows better results than DT and obtains better performance evaluation metrics for all classes.

The RF model is based on the structure of multiple decision trees. RF divides the dataset and creates the decision tree for each sample set. Each decision tree extracts the patterns according to the samples selected and performs predictions and extracts the results. In the latter, the RF performs voting operations between all decision trees and gives a prediction according to the averaging of all decision trees. The multiple tree-based architectures enhance the classification predictions that allow the detection of threats more effectively. Despite poor performance by the DT model, RF shows superior results for the experiments carried out in this study.

SVM leverages the concept of hyperplane and divides the data into hyperplanes and tries to increase the distance between class margins. According to the straight hyperplane, the classification of the outlier values or threats is difficult to detect. Then a kernel is used by the SVM to convert the non-linear hyperplane by converting the two-dimensional space into three-dimensional space, which helps to enhance its performance. In this study, RF shows better performance than the other models employed for the experiments.

RF divides the dataset and creates the decision tree for each sample set. In the latter, RF performs voting operations for the final prediction. The multiple tree-based architecture enhances the classification predictions that allow the more effective detection of threats. In previous studies, RF was also proven to show superior performance for network attack detection. Similarly, although KNN is a lazy learner, it has been proven to be better than many complex models for network attack detection. It shows better performance when the training data are noisy. Moreover, the number of training samples is large, which is suitable for KNN.

SVM divides the data into hyperplanes and tries to increase the distance between class margins. It is suitable when the data are high-dimensional, which is the case with the dataset used in this study. RF can also handle high dimensional space very well, in addition to a large number of training samples. Consequently, when these algorithms are joined as an ensemble, they show better performance.

The proposed ensemble RKSVM model combines three individual models to enhance threat prediction efficacy. The ensemble models, in which three machine learning models are merged together to perform classification, tend to show better performance than individual models. In the case of network threat detection, the performance of the proposed RKSVM is also better than the other models. The accuracy results are shown in Table 8, and the highest results are achieved with the class DoS, with an accuracy of 99.8%, precision 99.8%, recall 99.6%, and F-measure 99.7%.

Security is a big issue in the IoT around the world among the many IoT devices. Security challenges have developed through the rapidly increasing technologies and the low knowledge about the advanced challenges that cause these problems of security. Some regulatory changes and profound security challenges are also involved in the security concerns. Concerns for technical security are similar to smartphones, workstations, and conventional servers, and these include forgetting about the default credentials changing, weak authentication, the sending of unencrypted messages in the different devices, poor handling of security updates, and SQL injections. The application of the proposed research will be implemented in eldercare, healthcare and medicine, home and building automation, industrial applications, manufacturing and agriculture, and military applications. Moreover, the proposed approach can be implemented in the industrial IoT sector. The implementation needs Raspberry Pie as a mini microprocessor that can store the trained model and can control the IoT sensor-based network communication.

### 4.3. Validation of Proposed Approach

To further verify and validate the performance of the proposed approach, experiments were performed using two additional datasets, the KDD dataset and the CIC-IDS 2017 dataset, that contain the same classes for network attacks. These benchmark datasets were utilized for the validation of the proposed IoTTPS system. Experimental results for these experiments are provided in Table 9. It shows the accuracy of network attack detection of the proposed model. The results indicate that the model performs well for other datasets as well, which shows its generalizability. The NSL-KDD dataset presents higher results than the KDD and CIC-IDS 2017 datasets. The results for the KDD dataset are slightly poorer than for other datasets; however, similar results are reported for the KDD dataset in the existing literature.

### 4.4. Computational Complexity of Models

To analyze the computational complexity of the proposed approach and other models used in this study, the detection time, throughput time, and latency of all models are reported. These parameters are used with the definitions given in Table 10.

The computational complexity of the models is given in Table 11. The results indicate that the proposed model has high computational complexity, which is due to its ensemble nature where multiple models are combined to obtain a better threat detection accuracy. We intend to reduce its computational time in the future.

### 4.5. Comparison with Existing Approaches

Table 12 illustrates the comparative analysis of the proposed approach with existing models. For this purpose, several existing models were selected. Only those studies that utilized the same dataset were selected so that a fair comparison could be made. Performance comparison indicates that the proposed approach outperforms existing models. The proposed IoTTPS system with the utilization of the NSL-KDD dataset illustrates the highest threat detection results.

The IOTTPS system has been created to detect threats in IoT networks. The major goal of this research is to build a system based on machine learning and increase threat detection accuracy. The performance of the machine learning models is analyzed in this research but still does not achieve much higher results with a single model. Instead of moving toward deep learning or more complex algorithms, the efficiency of the machine learning algorithms is increased in this research. When the machine learning models are joined to make an ensemble, time complexity may become higher than individual models because all three model needs time to produce predictions. However, the accuracy is enhanced compared to individual models. Thus, traditionally, it is a trade-off between accuracy and computational complexity. For applications where enhanced accuracy is important such as military systems and government systems, the proposed approach is best suited as it provides much better results than other complex and low-accuracy-based systems [72,73,74].

## 5. Conclusions

IoT technology is growing very rapidly and becoming a part of the user’s daily life. Due to its heterogeneous nature, an IoT network is susceptible to a large number of network threats. The threat analysis in the IoT network is performed in this study using the proposed IoTTPS system. Experiments are performed using the NSL-KDD dataset that contains 23 different types of threats, which are categorized into four major categories, DoS, Probe, R2L, and U2R. Experiments involve the implementation of the proposed ensemble RKSVM model and several well-known machine learning models, DT, NB, LR, RF, SVM, GBM, and KNN. The results demonstrate that the highest threat detection accuracies achieved with the ensemble RKSVM model based on grid search and cross-fold validation are 99.7%, 99.3%, 99.7%, and 97.8% for DoS, Probe, U2R, and R2L attacks, respectively. This research aims to detect the threats in the IoT network, which is successfully achieved. The enhancement in security based on the proposed research and the prevention of cyber-attacks will enable users to trust the use of their IoT devices in daily life.

Deep learning and transfer learning algorithms with genetic algorithms will be implemented in the future to provide security in bigger networks. The major limitation of this research is the time complexity. The ensemble RKSVM model is based on three machine learning models. Every model in this ensemble method needs to produce a prediction to apply an averaging method to calculate the final output prediction on test data, which increases the time complexity of the proposed model. The transfer learning and deep learning models will be used to create a model that will produce a prediction much faster and more efficiently. We also intend to use Bayesian optimization to find the optimal hyperparameter for the ensemble model in our future work. 

## Figures and Tables

**Table 1:** Comparative literature analysis.

Ref.	Summary	Features	Evaluation
[40]	Entropy-based early detection mechanism of DDOS attack and flash events.	Packet header, time window size, and other generalized parameters. Dataset from CAIDA, MIT Lincoln, and FIFA	F measure, precision, false positive rate, and accuracy
[41]	Detection mechanism to detect a flow-table attack, bandwidth attack, and controller attack in SDN environment using machine learning techniques and scapy tool for attack simulation.	Byte_count, flow alive in nanoseconds, port ID, type of service, maximum length to send the controller.	Accuracy, time to process.
[42]	Four behavioral features show specific behavior to differentiate normal and malicious traffic.	The dataset is from Clarknet, worldcup98, and NASA	False positive, false negative, true positive, true negative.
[43]	Hadoop-based real-time detection scheme to detect DDOS traffic using Map Reduce and HDFS	Experimental dataset based on Source IP, destination IP, packet protocol, timestamp, and packet header.	CPU utilization and memory
[44]	URL entropy-based DOS detection algorithm was used to analyze attack traffic and a mapping matrix of joint entropy vector is contracted.	MIT Lincoln dataset based on source IP address, time window size, and other generalized parameters.	Space complexity, relative strength, time
[45]	Bio-inspired approach to detect HTTP flood attack with minimal process complexity.	Dataset of CAIDA based on minimum time interval, number of sessions, and page access count.	Recall, precision, true positive, false positive, true positive, true negative.
[46]	An LSTM network model is used to analyze malicious and legitimate traffic using H-ping tool for simulation	ISCX 2012, CTU 13, and experimental dataset	Accuracy, error rate
[47]	Asymmetric detection mechanism is designed by annotated probabilistic timed automata and suspicion scoring algorithm to recognize the DDOS traffic.	Existing server logs, access traces, think time. Experimental dataset.	Precision, F1 measure, detection, false positive, false negative

**Table 2:** Major categorization of attacks into classes.

Classes	Numeric Representation
Normal	0
Dos	1
Probe	2
Remote-to-Local (R2L)	3
User-to-Root (USR)	4

**Table 3:** The class-wise dataset presentation according to classes in training and testing data.

Classes	Training Data Dimensions	Testing Data Dimensions
Dos	113,270	17,171
Probe	78,999	12,132
R2L	68,338	12,596
USR	67,395	9778

**Table 4:** Best parameters extracted from grid search for models used for ensemble.

Models	Parameter	Values
RF	n_estimators	10
	n_jobs	2
	criterion	Gini
KNN	n_neighbors	5
	leaf_size	30
	P	2
	metric	Minkowski
SVM	kernal	linear
	C	1.0
	random_state	0

**Table 5:** Experimental results of models in terms of accuracy.

Classes	DT	NB	LG	GB	RF	SVM	KNN	IoTTPS
DoS	89.2	86.7	99.4	99.5	99.7	99.3	99.7	**99.7**
Probe	96.5	97.8	98.3	98.7	99.2	98.4	99.0	**99.2**
U2R	99.5	97.2	99.6	99.5	99.6	99.6	99.7	**99.7**
R2L	87.3	93.5	96.5	97.2	97.1	96.7	96.7	**97.2**

**Table 6:** Comparative performance analysis of proposed approach and machine learning models in terms of precision.

Classes	DT	NB	LG	GB	RF	SVM	KNN	IoTTPS
DoS	88.0	98.8	99.1	99.2	99.7	99.1	99.6	**99.8**
Probe	92.7	97.3	97.04	97.4	98.3	96.9	98.6	**98.7**
U2R	90.6	60.1	93.0	87.7	94.2	91.0	93.1	**94.3**
R2L	87.55	89.0	94.4	95.3	96.3	94.8	95.3	**95.8**

**Table 7:** Performance analysis of proposed approach and several machine learning models in terms of recall.

Classes	DT	NB	LG	GB	RF	SVM	KNN	IoTTPS
DOS	87.8	70.3	99.5	99.6	99.6	99.4	99.6	**99.6**
Probe	97.5	96.0	97.9	98.8	98.7	98.3	98.5	**98.9**
U2R	72.7	97.9	83.7	80.6	84.5	82.9	85.0	**86.5**
R2L	74.9	95.5	96.0	95.6	96.3	96.2	95.4	**96.2**

**Table 8:** Comparative analysis of proposed approach and several machine learning models in terms of F1 score.

Classes	DT	NB	LG	GB	RF	SVM	KNN	IoTTPS
DoS	87.5	82.1	99.3	99.4	99.7	99.2	99.6	**99.7**
Probe	94.8	96.6	97.4	98.1	98.9	97.6	98.5	**98.7**
U2R	78.0	66.0	86.4	82.1	85.1	84.8	87.8	**89.1**
R2L	78.8	91.6	95.2	96.0	96.9	95.5	95.3	**96.0**

**Table 9:** Comparative analysis of proposed IoTTPS on multiple datasets.

Parameter	Classes	NSL-KDD Dataset	KDD Dataset	CIC-IDS 2017 Dataset
Accuracy	DOS	99.7	93.4	99.3
	Probe	99.2	96.6	97.4
	U2R	99.7	92.0	94.6
	R2L	97.2	91.6	95.2
Precision	DOS	99.8	82.1	99.3
	Probe	98.7	97.2	95.8
	U2R	94.3	86.7	89.1
	R2L	95.8	93.9	91.3
Recall	DOS	99.6	94.3	96.7
	Probe	98.9	97.3	95.3
	U2R	86.5	92.4	89.9
	R2L	96.2	93.4	95.2
F1 score	DOS	99.7	82.1	99.3
	Probe	98.7	96.6	96.4
	U2R	89.1	64.0	86.4
	R2L	96.0	97.6	96.2

**Table 10:** Units used for computational complexity.

Metrics	Units
Detection Time	Time (seconds) taken by model to predict testing data based on threats
Throughput	Number of threats detected per second
Latency	Time (seconds) taken by detection of the single attack

**Table 11:** Computation cost of the proposed approach and machine learning models with NSL-KDD dataset.

Models	Classes	Detection Time	Throughput	Latency
GBM	DOS	0.126007	136,270.1206	0.0156266
	Probe	0.0370032	327,862.83852	0.016377
	R2L	0.0330026	381,666.7257	0.0260016
	U2R	0.0240008	407,401.6759	0.0156264
DT	DOS	0.0170013	1,009,976.2159	0.015991
	Probe	0.011001	1,102,797.8008	0.0156259
	R2L	0.01200175	1,049,513.36307	0.01562595
	U2R	0.01000189	977,614.4671	0.0157036
NB	DOS	0.2400138	71,541.70841	0.0161867
	Probe	0.0830039	146,161.6346	0.0159137
	R2L	0.092005	186,631.1321	0.01562643
	U2R	0.0710043	137,709.7937	0.0158147
LR	DOS	0.0130021	1,320,627.0098	0.01770687
	Probe	0.00800156	1,516,203.2158	0.0159666
	R2L	0.0140025	899,549.6957	0.01583218
	U2R	0.0070016	1,396,530.2724	0.038001775
RF	DOS	0.038001	451,852.9759	0.32101
	Probe	0.041002	295,887.7518	0.24401
	R2L	0.030002	419,837.0379	0.06249
	U2R	0.021001	465,578.7906	0.060003
KNN	DOS	881.4291	19.48086	0.046877
	Probe	645.59371	18.792004	0.046875
	R2L	489.99926	25.70616	0.04687
	U2R	379.42463	25.77059	0.038002
SVM	DOS	2.379104	7217.4211	0.015735
	Probe	3.45194	3514.54465	0.015835
	R2L	2.25552	5584.5189	0.060129
	U2L	0.203546	48,038.2138	0.015887
Ensemble RKSVM	DOS	859.04686	19.98843	0.062499
	Probe	599.36881	20.24129	0.0468778
	R2L	515.34198	24.44202	0.046875
	U2R	379.880803	25.73965	0.046874

**Table 12:** Comparative analysis of proposed approach with previous approaches.

Ref.	Year	Dataset	Model	Accuracy	Precision	Recall	F1-Score
**IoTTPS**	2023	NSL-KDD	Ensembled RKSVM model	99.7%	99.8%	99.6%	99.7%
[62]	2022	NSL-KDD	Multilayer Perceptron	97.6%	97.9%	67.3%	-
[63]	2022	NSL-KDD	RF	-	99.4%	99.3%	99.6%
[64]	2021	NSL-KDD Test+	Hybrid classifier	85.2%	86.5%	85.2%	84.9%
[65]	2021	NSL-KDD	DSSTE-AlexNet	82.8%	83.9%	82.7%	81.6%
[66]	2020	NSL-KDD	AESMOTE	82.0%	-	-	82.4%
[67]	2021	NSL-KDD	I-SiamIDS	80.0%	-	-	68.3%
[68]	2020	NSL-KDD	TIDCS	98.0%	-	-	-
[69]	2019	NSL-KDD	Adaboost classifier	93.4%	96.1%	91.4%	93.7%
[70]	2019	NSL-KDD	Multi tree classifier	84.23%	86.4%	84.23%	83.6%
[71]	2019	NSL-KDD	AE-RL	80.1%	-	-	79.4%

## Data Availability

Data is available on a reasonable request from authors.
